# A comprehensive comparison between young and older-age non-small cell lung cancer patients at a public referral centre in Delhi, India

**DOI:** 10.3332/ecancer.2021.1223

**Published:** 2021-04-27

**Authors:** Vishal Vashistha, Avneet Garg, Hariharan Iyer, Deepali Jain, Karan Madan, Vijay Hadda, Randeep Guleria, Anant Mohan

**Affiliations:** 1 Raymond G. Murphy New Mexico Veterans Affairs Healthcare System, Section of Haematology and Oncology, 1501 San Pedro Dr SE, Albuquerque, NM 87108, USA; 2 Department of Pulmonary Medicine and Sleep Disorders, All India Institute of Medical Sciences, New Delhi 110608, India; 3 Fulbright-Nehru Fellowship Programme, United States-India Educational Foundation, Fulbright House, 12 Hailey Rd, New Delhi 110002, India; 4 Adesh Institute of Medical Sciences and Research, Department of Pulmonary Medicine, Barnala Bypass, Bathinda 151109, India; 5 Department of Pathology, All India Institute of Medical Sciences, Sri Aurobindo Marg, Ansari Nagar, Ansari Nagar East, New Delhi 110029, India

**Keywords:** lung cancer epidemiology, young cancer patients, non-smokers with lung cancer, adenoid cystic carcinoma

## Abstract

**Purpose:**

Given the increasing number of non-small cell lung cancer (NSCLC) patients in India, a comparative analysis between patients under 40 years and those of older age at a major public referral centre would provide insight into the phenotypic patterns of this group.

**Methods:**

NSCLC patients who were accessioned within the lung cancer clinic database of the Pulmonary Medicine Department at the all India institute of medical sciences – Delhi between 2008 and 2019 were reviewed. Patients 40 years or younger and 60 years or older were selected and categorised as young and older patients, respectively. Baseline clinical characteristics, histologic profiles, treatments administered and survival outcomes were compared between both groups.

**Results:**

Following the database review, 154 young and 1,058 older patients were selected for inclusion. Clinically, young patients were more often female (26.0% versus 14.5%, *p* < 0.001), retained a more independent performance status (64.1% versus 45.5%; *p* < 0.001) and never smoked (63.7 % versus 18.8%, *p* < 0.001). Regarding disease profiles, young patients were more frequently diagnosed with adenocarcinoma (*p* < 0.001) and 12 young patients had adenoid cystic carcinoma. Rates of stage IV disease at presentation were higher among young patients (78.0% versus 63.0%, *p* < 0.001). Regarding treatment, no differences in systemic therapies administered or survival were identified.

**Conclusion:**

In India, young NSCLC patients are frequently non-smokers and diagnosed with advanced disease. Despite better performance status, young patients do not share better outcomes. Efforts should be directed towards optimising intensive treatment for young patients.

## Introduction

Lung cancer remains the leading contributor to cancer-related mortality worldwide [1]. Considerable optimism has grown with the development and availability of targeted therapies and check point inhibitors, particularly for patients with metastatic non-small cell lung cancer (NSCLC). These precision therapies extend survival outcomes and occasionally provide durable responses primarily for select patients with favourable molecular profiles and/or expression of tumour biomarkers [2–4]. Nevertheless, the 5-year overall survival (OS) for patients with locally advanced or metastatic NSCLC is still poor [5]. As a result, young patients with lung cancer remain an exceedingly concerning group for providers, despite the expansion of treatment options in recent years.

The disease features and treatment outcomes among young NSCLC patients in the US, Western Europe and East Asia have been frequently studied [6–15]. Commonly, these comprehensive investigations of single-institutional or multi-centre cancer databases have sought (1) to quantify the rates of driver mutations and/or germline mutations among those with advanced NSCLC and/or (2) to recognise opportunities to explore more aggressive multimodality treatment regimens among those with early-stage diseases. Insight into potentially higher risk groups of young patients, including those of lower socio-economic status and young patients bearing unique disease histology, has often been neglected. In addition, to our knowledge, the Indian experience of young patients with NSCLC is yet to be reported.

With India’s massive population, growing number of tobacco users and improving delivery of healthcare services, the number of young Indian patients diagnosed with NSCLC is expected to rise [16, 17]. Compared to patients of older age, young patients may present unfamiliar challenges to clinicians. Indian providers will increasingly be tasked with managing clinical expectations based upon previously reported outcomes, administering aggressive treatment regimens and navigating through the emotional complexities associated with young patients harbouring a frequently terminal disease. Additionally, the unique findings from the relatively larger number of young patients with NSCLC in India could provide critical information regarding this rare group of patients elsewhere. Consequently, a comprehensive evaluation addressing the clinical features, disease profiles, treatments administered and survival outcomes among young NSCLC patients in India is warranted.

The primary aim of this study was to compare the clinicopathological features and treatment experience between young patients and older patients with NSCLC attending a public tertiary referral centre in north India. We have previously shown that the rate of advanced-stage disease is markedly high at our centre [18]. Given that providers are often ambivalent towards mild or moderate respiratory symptoms among younger patients, we proposed the following hypotheses: (1) the rate of metastatic disease would be similar to or potentially higher among young patients compared to older patients; (2) the rate of adenocarcinoma would be higher among young patients, and consequently, the rates of actionable mutations would be higher as detailed in previous reports and (3) the performance status among young patients would be better, which in turn would lead to improved treatment outcomes compared to older patients [6, 7, 10, 11].

## Methods

### Included subjects

Patients who (1) had a histologic diagnosis of NSCLC confirmed by a thoracic pathologist between January 2008 and December 2019, (2) underwent sufficient clinical and staging evaluation (3) and were either 40 years or younger or 60 years or older were selected. With regard to sufficient clinical and staging documentation, the selected patients were required to have baseline demographic data and results of initial baseline staging scans readily available. Patients with a diagnosis of small cell lung cancer or insufficient baseline clinical characteristics and/or histological details were excluded. Patients who were 40 years or younger were categorised as ‘young patients’. Patients who were 60 years or older were categorised as ‘older patients’. The selection of age groups was based upon two sources of information. First, prior evaluations conducted in other countries of young patients with lung cancer frequently examined those younger than 40 or 45 years of age [6, 9, 11, 12]. Second, we previously reported that the mean age of lung cancer patients at our centre was approximately 59 years [19]. The lower limit of our older cohort was chosen to be higher than the mean age of patients managed at our site.

### Data source and ethical considerations

Eligible patients were initially selected from the Lung Cancer Clinic (LCC) database of the Pulmonary Medicine Department of the All India Institute of Medical Sciences (AIIMS) in Delhi, India. This database includes all patients who have undergone evaluation, diagnostic investigation and/or treatment for lung cancer within the LCC. Each unique record in the database provides possible entries for (1) patient demographics; (2) disease characteristics, including histological details; (3) imaging assessments; (4) treatments administered and (5) clinical outcomes. As a public tertiary referral centre, AIIMS predomi nantly serves lower income patients in Delhi and the National Capital Region. The vast majority of patients have metastatic disease upon presentation, and consequently, the rates of surgery and radiation therapy with curative intent are exceedingly low [18].

This study was formally approved by the Institutional Ethics Committee within the Research Section at AIIMS (IEC/536/9/2018). Being a retrospective evaluation, consent was not required from included subjects.

### Data collection and analysis

The baseline clinical and disease characteristics, histological profiles, imaging assessments, treatments administered and outcomes were abstracted from the LCC database. Baseline imaging was frequently conducted by either computed tomography (CT) with or without intravenous contrast or positron emission testing (PET) near the time of diagnosis. Follow-up imaging was conducted by PET at 3-month intervals if treatment(s) was administered. If available, mutational data within the epidermal growth factor receptor (*EGFR*) or anaplastic lymphoma kinase (*ALK*) genes were obtained.

Demographic details, including age, gender, monthly income and smoking status, baseline disease characteristics and treatments administered were compared between young and older patients. Treatments included systemic therapy, which herein encompassed traditional cytotoxic chemotherapy in addition to targeted treatments for eligible patients, radiation therapy with or without curative intent and surgery. Categorical variables were compared using chi-square testing; means were compared using *t*-testing; and medians were compared using Mood’s median test. Differences were deemed significant at *p* < 0.05.

Treatment outcomes were compared between young and older patients based upon objective response rate (ORR), disease control rate (DCR), progression-free survival (PFS) and OS. ORR and DCR were strictly determined by reviewing the imaging reports for each patient who underwent treatment. The review of imaging reports relied upon radiologists’ interpretations of changes in sizes of tumours or number of tumours. PFS was computed for each patient using the date of treatment initiation and date of progression, which was based upon imaging, clinical progression or death. Among patients without progression, dates of last contact were included, and these patients were censored for PFS analysis. Among patients who remain alive, the dates of last contact were included and these patients were censored for OS analysis. Kaplan–Meier curves were constructed and univariate PFS and OS were compared using log-rank testing. A multivariate Cox proportional hazards model was additionally designed. In addition to comparing young patients with older patients, the model parameters included sex, baseline performance status, history of smoking, stage of disease, histology and presence of actionable mutations.

## Results

Following the review of the LCC database, which included 2,427 total lung cancer patients and 2,072 patients with NSCLC, 154 young patients and 1,058 older patients were selected for inclusion. The mean (SD) age was 35.2 (4.8) years and 66.5 (5.9) years for young and older patients, respectively. A trend towards increased visits of young patients at our LCC was observed over time compared to older patients (*p* = 0.052), as shown in Table 1

### Clinical features of young and older patients

Compared to older patients, young patients were more likely to be female (26.0% versus 14.5%, *p* < 0.001), non-smokers (63.7% versus 17.8%, *p* < 0.001), receive secondary education or higher (46.1% versus 37.6%, *p* = 0.042), be unemployed (40.0%, versus 31.3%,* p* = 0.020) and have an Eastern Cooperative Oncology Group (ECOG) performance status of 0 or 1 (64.1% versus 45.5%, *p* < 0.001). No differences were observed in the median duration of symptoms prior to presentation to our LCC, monthly median income or family history of malignancy between young and older patients.

### Differences among disease characteristics and treatments administered

The differences among disease profiles and treatments administered are presented in Table 2. Regarding histology, young patients were more often diagnosed with adenocarcinoma compared to older patients (53.9% versus 36.6%, *p* < 0.001). As expected, older patients were more frequently diagnosed with squamous cell carcinoma (14.9% versus 41.2%,* p* < 0.001). Among the 23 young patients who were diagnosed with squamous cell carcinoma, 15 (65.2%) reported of never smoking previously.

**Figure 1.**
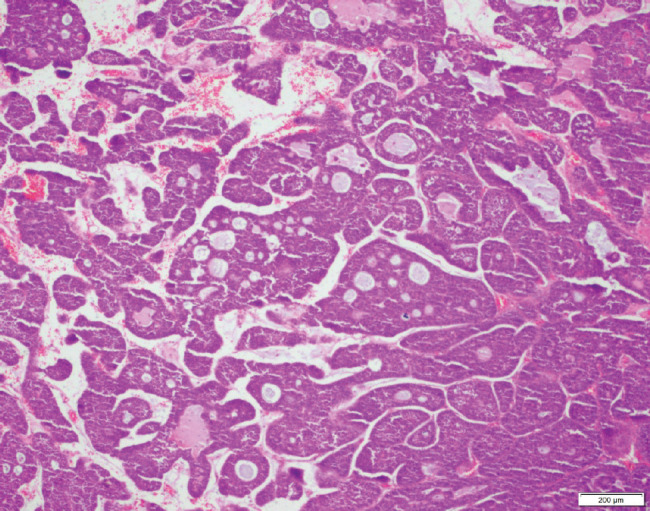
Representative tissue sample of ACC extracted from a young patient attending our LCC. The photomicrograph conveys tumour cells arranged in a cribriform pattern. The cystic spaces are filled with slight basophilic material. Upon further magnification, scant cytoplasm was identified in most cells, which is a classic feature of the disease.

Twelve young patients were diagnosed with adenoid cystic carcinoma (ACC), an uncommon salivary gland-type malignancy that rarely develops along the central airways, whereas no older patients were given this diagnosis. A histologic slide from a tumour sample bearing this rare variant is shown in Figure 1. Although testing for actionable mutations was infrequent, trends towards higher rates of *EGFR* mutations (*p* = 0.093) and *ALK* rearrangements (*p* = 0.059) were observed for young patients. Actionable mutations were assessed by immunohistochemical staining (IHC) and/or quantitative polymerase chain reaction (QPCR) of tumour samples.

Regarding the stage of disease, young patients more frequently presented with distant metastases than older patients (78.0% versus 63.0%, *p* < 0.001). Nevertheless, rates of advanced disease, including both stage III and IV, were similar between the two groups (94.5% versus 94.8%). In turn, no differences between rates of systemic therapy (*p* = 0.613) or radiation therapy (*p* = 0.166) administrations were observed. Young patients were more likely to undergo surgery (*p* = 0.028) with the critical nuance that rates of surgical intervention were remarkably low for both groups.

### Comparison of treatment outcomes

Only 36 (50.0%) out of 72 young patients and 226 (43.4%) out of 521 older patients had received systemic therapy and underwent sufficient initial and follow-up imaging to evaluate DCR and ORR. No differences were observed in either DCR (*p* = 0.345) or ORR (*p* = 0.802) between the two groups of patients.

**Figure 2.**
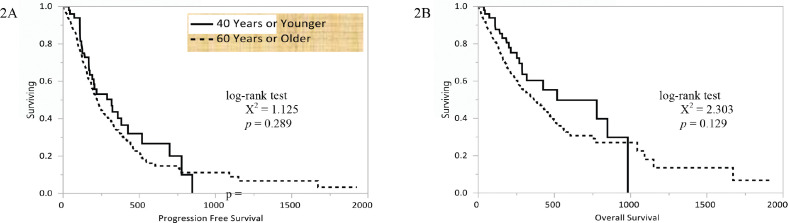
Kaplan–Meier curves for (a) PFS and (b) OS in days based upon age for patients who had received systemic therapy for NSCLC at our centre.

The Kaplan–Meier curves conveying the PFS of young and older patients are shown in Figure 2a. Young patients had a median PFS of 10.6 (4.9–23.3) months and older patients had a median PFS of 7.5 (6.6–8.6) months following the initiation of systemic therapy (*p* = 0.289). The Kaplan–Meier curves conveying the OS of young and older patients are shown in Figure 2b. Young patients had a median OS of 17.3 (8.5–32.7) months and older patients had a median OS of 12.2 (4.9–34.8) months following the initiation of systemic therapy (*p* = 0.129). Among 67 patients receiving targeted therapies for *EGFR* mutations, no differences in median PFS (6.9 versus 9.5 months, *p* = 0.669) or OS (unreached for both groups, *p* = 0.560) were observed.

The designed multivariate Cox proportional hazards model computed a hazard ratio (HR) of 0.62 (0.26–1.47) for PFS among young patients. Using the same model parameters, the HR for OS among young patients was 0.61 (0.21–1.81).

## Discussion

To our knowledge, this is the first comparison between young and older-age Indian patients with NSCLC. A trend towards an increased number of young patients was observed. However, given the presence of convoluting variables, namely the growing development of our LCC and the improving accessibility to primary care providers among patients in Delhi, we are unsure of the significance of this finding. Young patients are more likely to be female, maintain a better performance status, abstain from prior smoking and undergo secondary education. The disease histology of young Indian patients is more frequently consistent with adenocarcinoma. Interestingly, ACC was diagnosed exclusively in younger patients. Although young patients are clinically more robust upon presentation, no differences in outcomes with use of systemic therapy were observed. A significant number of young patients with adenocarcinoma (particularly before 2014) had not undergone testing for mutations within *EGFR* or *ALK* at our LCC, thereby mitigating potential improvements in survival with use of targeted treatments among a group that commonly shares actionable gene variants.

In the current study, the percentage of females was almost double among young patients compared to older patients. A major caveat is that the expected number of female patients was substantially lower for both young and older groups [20]. As we had recently investigated, the lower rates of women presenting with lung cancer at our centre is primarily due to the Indian healthcare system frequently misdiagnosing ongoing respiratory symptoms among non-smoking women as tuberculosis [21]. Nevertheless, it has recently been shown that the incidence of lung cancer is likely higher among young women compared to older-age women [6]. Clinicians must be wary of the malignancy for young women with unexplained respiratory symptoms regardless of their smoking status.

Our findings indicate that young Indian patients are more likely to have metastatic disease at the time of diagnosis. Although the percentages of all NSCLC patients with advanced disease are lower, previous studies in France, Japan and China indicate unexpectedly higher rates of stage III or IV disease among young patients compared to older patients [6, 10, 15]. In the US, Liu *et al* [11] reviewed the surveillance, epidemiology and end results database to compare disease characteristics of NSCLC patients between 18 and 30 years of age and 31 and 40 years of age. The authors interestingly observed that patients under 30 years had decreased rates of advanced lung cancer compared to the slightly older group. In summation, young patients are commonly staged with advanced disease upon diagnosis, which could be the result of ambivalence towards symptoms among patients and/or providers or more aggressive disease biology. Specific age groups among young patients may share differing rates of advanced disease.

The results from our study and that of previous investigations confirm that young patients with NSCLC have substantially lower rates of tobacco use [6, 7, 15]. In conjunction with our evaluation, studies have collectively observed that approximately one-half of young patients share a history of smoking, whereas the rate of smoking for all patients with NSCLC approaches 90% [22]. To better assess this stark contrast, Fritz and Olsson [14] administered questionnaires to young Swedish women with lung cancer. The authors found substantially high rates of second-hand exposure to tobacco, radiation exposure through prior imaging and family history of malignancy. As the number of young NSCLC patients with germline mutations is relatively low, similar future investigations detailing the role of environmental factors are critical [13]. Specifically, in a developing region, such as Delhi, India, with recent rapid industrial growth, concerns regarding air quality should be considered.

The higher prevalence of adenocarcinoma among young patients in our study is in accordance with the results of previous studies across the world [6, 7, 9, 10, 23]. Compared to other histologic types, adenocarcinoma is more frequently diagnosed in patients who have never used tobacco, which is consistent with the lower rates of smoking among younger NSCLC patients [24].

In conjunction with the higher rates of pulmonary adenocarcinoma, highly actionable gene variants within *EGFR*, *ALK*, and Reactive Oxygen Species Proto-Oncogene 1 (*ROS-1*) are more commonly identified in young patients based upon previous work. In fact, Sacher et al had observed that nearly 60% of young patients who had undergone genotyping at a major US centre had an actionable mutation in one of the aforementioned genes or B-rapidly accelerated fibrosarcoma (*BRAF*). Given that the overall rates of *EGFR* mutations are highest among south Asian patients with NSCLC, a large volume of young Indian patients are likely candidates for tyrosine kinase inhibitors [25]. Unfortunately, only 54.2% of young patients with adenocarcinoma at our LCC underwent testing by IHC and/or QPCR. A substantially lesser number of older patients underwent testing. As of now, the greatest contributors to lower testing rates at our site have not been defined, but it is likely that the socio-economic status of our patients frequently leads to fragmentation in care, particularly during the initial phase of tumour evaluation. As a result, we are working collaboratively with clinicians at our LCC, NSCLC patients and our pathology colleagues to ensure prompt clinic evaluations following tissue biopsies, streamline reflexive IHC, to initiate the use of our on-site next-generation sequencer and to investigate the delivery of therapies based upon the timely results of precision testing [26].

Nearly 8% of young patients in our study were uniquely diagnosed with pulmonary ACC, a histology that is far more frequently identified within tumours of the head and neck [27]. Salivary gland-type tumours are rarely diagnosed as lung cancers, and historically, mucoepidermoid carcinoma (MEC), fol lowed by ACC and epithelial–myoepithelial carcinoma, is most common [28, 29]. These tumours are considered low grade and are often diagnosed among younger patients with no prior smoking history. Salivary gland-type tumours of the lung are usually identified along the proximal airways and rarely in the periphery of the lungs. Once we were aware that only young patients harboured ACC, we reviewed the presence of MEC in our LCC database and identified one 80-year-old male previously harbouring this diagnosis. He was excluded from the selection in this study as integral components of his clinical background and disease data were unavailable. Further studies are needed to confirm the epidemiology of ACC among young Indian patients and to provide insight regarding optimal treatment approaches for this low-grade malignancy.

Unfortunately, given that nearly 80% of young patients at our LCC are diagnosed with metastatic disease upon presentation, an evaluation of aggressive multimodal treatment regimens within this group was not feasible. This is largely a reflection of our patients facing difficulties with returning for appointments frequently. Specifically, rates of ‘curative’ interventions, namely surgery and radiotherapy for patients with early-stage disease, are unfortunately low. Methods to improve the delivery of care to our predominantly lower income population are being evaluated. Regardless, we did observe a small increase in rates of surgery among young patients compared to older patients. Two out of three prior related studies have indicated that young patients with early-stage NSCLC often undergo more intensive treatment regimens, while Jiang *et al* [7]. actually observed a lower rate of surgeries among young patients in their single-centre retrospective analysis [8, 10]. In their comprehensive evaluation of nearly 180,000 patients in the U.S. National Cancer Database, Arnold *et al* [8] had observed that NSCLC patients under 47 years old had underwent more aggressive therapy for each stage of disease compared to patients above 47 years of age. Unfortunately, only minimal improvement in 5-year survival was found among young patients per each disease stage, which suggests that either intensive therapies are not more effective, or the disease biology of young patients is more aggressive. To our knowledge, no clinical trials have assessed the role for more intensive treatments for young NSCLC patients, which represents a significant gap in the current literature particularly as curative multimodal treatment options for oligometastic disease are becoming more apparent. Additionally, we were encouraged to find that discrimination against older patients regarding rates of standard systemic therapy administrations was not observed at our LCC.

No differences in survival were observed between young and older patients receiving systemic therapy in our study. The previously reported survival outcomes with systemic therapy for young patients have been mixed, with some studies confirming improved survival and others showing no differences compared to older patients [7–9, 12, 15]. Interestingly, in the work by Sacher *et al* [12] which evaluated outcomes within five different age groups based upon the presence of actionable mutations, no survival difference was observed between patients under 40 years and those over 70 years with use of targeted treatments. The authors had concluded that the lack of improvement in survival among younger patients clearly suggested that their disease biology was more aggressive. Facing less comorbidities and more physiologic robustness, young NSCLC patients would be expected to live longer than older patients, especially with the presence of driver mutations being controlled. Similarly, our initial hypothesis that young patients would have better treatment outcomes at our LCC was incorrect, thereby providing further evidence to substantiate the notion that young patients may harbour more aggressive disease.

### Limitations

Our single-centre retrospective evaluation of predominantly lower income, young and older-age NSCLC Indian patients clearly carried a few major limitations. First, being a single-centre evaluation, our results may not be generalisable or extrapolated to other cancer centres internationally, or even to other centres in India which may be operating within dramatically different settings. Second, although we observed differences in rates of tobacco use between young and older patients, data regarding passive smoking were not readily available, and younger patients may have had more exposure to tobacco use than originally believed. Third, as the vast majority of patients attending our LCC are diagnosed with advanced disease, we were unable to measure the impact of radiation therapy and/or surgery on young patients with NSCLC. Last, given that our rates of precision testing were admittedly low even among patients with pulmonary adenocarcinoma, survival outcomes across both age groups in our study are likely lower than those at other centres with more developed precision programmes.

## Conclusion

From this phenotypic comparison, young Indian patients are more frequently female and staged with metastatic disease at time of diagnosis. Interestingly, almost 8% of young patients at our centre were diagnosed with ACC, a variant of primary salivary gland-type lung cancer. Despite maintaining a better performance status before treatment initiation, young NSCLC patients share similar responses and survival to systemic therapies with older patients. Such findings indicate that young patients harbour more aggressive disease biology. Efforts should be directed towards identifying the barriers to expediting the diagnosis of NSCLC among young patients, and thereby reducing the number of patients with metastatic disease, and prospectively evaluating the feasibility and responses with administering more intensive multimodal treatment regimens to young patients.

## Conflicts of interest

All authors have no conflicts of interest to disclose.

## Funding

The United States-India Educational Foundation, Fulbright-Nehru Fellowship Programme provided financial support to author VV to complete this work. This support included travel and accommodation.

## Authors’ contributions

VV, RG, and AM conceived the study concept. VV, AG, HI, DJ, KM, VH, and AM collected the study’s data. VV, AG, and AM completed the data analyses. All authors discussed the results and contributed to the final manuscript.

## Figures and Tables

**Table 1.** Baseline demographics for included patients.

	40 years or younger (*n* = 154)	60 years or older (*n* = 1,058)	
Mean age (SD)	35.2 (4.8)	66.5 (5.9)	*p* < 0.001
Females	40 (26.0%)	153 (14.5%)	*p* < 0.001
Year of diagnosis			
2008–2010	10 (6.5%)	71 (6.7%)	
2011–2013	24 (15.6%)	137 (12.9%)	*p* = 0.052
2014–2016	69 (44.8%)	379 (35.8%)	
2017–2019	51 (33.1%)	471 (43.1%)	
Median duration of symptoms^a^ [IQR]	120 [88.5–270] days	120 [90–210] days	*p* = 0.981
Smoking status			
Never	93 (63.7%)	180 (17.8%)	
Former	32 (21.9%)	329 (32.5%)	*p* < 0.001
Active	21 (14.4%)	502 (49.7%)	
Education			
Primary or less	83 (53.9%)	655 (62.4%)	*p* = 0.042
Some secondary or more	71 (46.1%)	394 (37.6%)	
Occupation			
Professional^b^	51 (44.4%)	492 (58.0%)	
Laborer/farmer	18 (15.7%)	91 (10.7%)	*p* = 0.020
Retired/unemployed	46 (40.0%)	266 (31.3%)	
Median monthly income [IQR]	6,000 [3,750–10,000] Rs	5,000 [3,000–10,000]	*p* = 0.499
Performance status			
ECOG 0 or 1	82 (64.1%)	409 (45.5%)	*p* < 0.001
ECOG 2 or greater	46 (36.0%)	490 (54.5%)	
Family history of malignancy	7 (5.2%)	39 (4.2%)	*p* = 0.565

SD = Standard deviation; IQR = Interquartile range; Rs = Rupees; ECOG = Eastern cooperative oncology group

a Duration of symptoms refers to the subjective interval of time between symptom onset and first visit with a lung cancer specialist at our center

b Professional occupation refers to positions requiring greater than secondary education to maintain

**Table 2.** Diagnostic and management differences.

	40 years or younger (*n* = 154)	60 years or older (*n* = 1,058)	
Histologic diagnoses			*p* < 0.001
Squamous cell carcinoma	23 (14.9%)	436 (41.2%)	
Adenocarcinoma	83 (53.9%)	387 (36.6%)	
NSCLC NOS	23 (14.9%)	217 (20.5%)	
Adenoid cystic	11 (7.1%)	0 (0%)	
Neuroendocrine tumor	5 (3.3%)	4 (0.4%)	
Other	9 (5.8%)	14 (1.3%)	
Stage of disease			*p* < 0.001
I	5 (3.9%)	12 (1.2%)	
II	2 (1.6%)	39 (3.9%)	
III	21 (16.5%)	314 (31.8%)	
IV	99 (78.0%)	622 (63.0%)	
Presence of actionable mutations^a^			
*EGFR* mutation	17 (37.8% of 45 patients tested)	47 (25.3% of 186 patients tested)	*p* = 0.093
*ALK* rearrangement	7 (23.3% of 30 patients tested)	11 (10.2% of 108 patients tested)	*p* = 0.059
Treatments administered			
Systemic therapy	72 (47.0%)	521 (49.2%)	*p* = 0.613
Cytotoxic agents	50	465	
Targeted therapies	20	54	
Both	2	2	
Radiation therapy^b^	14 (9.1%)	65 (6.1%)	*p* = 0.166
Surgery	6 (3.9%)	15 (1.4%)	*p* = 0.028
Initial treatment delivered within 2 weeks of diagnosis^c^	48 (67.6% of 71 patients)	355 (68.8% of 516 patients)	*p* = 0.839
PET/CT imaging response ratesd			
ORR	22 (61.1% of 36 patients)	119 (52.7% of 226 patients)	*p* = 0.345
DCR	27 (75.0% of 36 patients)	165 (73.0% of 226 patients)	*p* – 0.802

NSCLC = Non-small cell lung cancer; NOS = not otherwise specified; ORR = objective response rate; DCR = disease control rate

a At our site, a vast majority of patients evaluated for actionable mutations carry a diagnosis of metastatic lung adenocarcinoma

b Radiation Therapy includes treatment with curative or palliative intent

c Patients were evaluated for the interval of time required to initiate treatment if (1) they had received some form of treatment and (2) the dates of diagnosis and initial treatment were available

^d^Patients were evaluated for imaging-based response assessments if (1) they had received systemic therapy and (2) PET and/or CT imaging were conducted at baseline and 4–6 cycles following treatment initiation. Of note, PET/CT is the standard modality for evaluating response at our LCC
